# Supplementary material for: Association of risk management practices and financial performance of microfinance institutions in Ethiopia: A two-step system generalized method of moments approach
Source: PLoS One. 2025 Jul 10;20(7):e0321415. doi: 10.1371/journal.pone.0321415 (PMC12244555; doi:10.1371/journal.pone.0321415)
Supplement: Attachment — Submitted filename: PONE-D-24-16639_Editor.docx [file pone.0321415.s002.docx]

**Referee report for**

PONE-D-24-16639
TITLE: ASSOCIATION OF RISK MANAGEMENT PRACTICES AND FINANCIAL PERFORMANCE OF MICROFINANCE INSTITUTIONS IN ETHIOPIA

**To the Editor**

Dear Editor,

Thanks for allowing me to review this interesting article.

It analyzes how Microfinance institutions provide financial services to low-income clients, especially rural women, and have grown significantly to compete with formal banks. The author exploits a change in legislation promoted by Ethiopia's National Bank occurred in 1996, to evaluate the relationship between risk management practices and the financial performance of 24 Ethiopian MFIs from 2013 to 2023.

Compared to the existing literature, the author offers a comprehensive view of the risks affecting MFIs’ management practices, over a relatively extended period, and integrating the regulatory context in the risk analysis.

I found that there are some notable contributions and potential new aspects of this research, which I detail in my report to the Author, as well as main and minor concerns.

I will be happy to revise further versions of the manuscript.

Best regards,

Lucia Dalla Pellegrina
