# Supplementary material for: Association of risk management practices and financial performance of microfinance institutions in Ethiopia: A two-step system generalized method of moments approach
Source: PLoS One. 2025 Jul 10;20(7):e0321415. doi: 10.1371/journal.pone.0321415 (PMC12244555; doi:10.1371/journal.pone.0321415)
Supplement: Attachment — Submitted filename: PONE-D-24-16639_Authors.docx [file pone.0321415.s003.docx]

**Referee report for**

PONE-D-24-16639
TITLE: ASSOCIATION OF RISK MANAGEMENT PRACTICES AND FINANCIAL PERFORMANCE OF MICROFINANCE INSTITUTIONS IN ETHIOPIA

**To the Authors**

This paper analyzes how Microfinance institutions provide financial services to low-income clients, especially rural women, and have grown significantly to compete with formal banks. The authors exploit a change in legislation promoted by Ethiopia's National Bank occurred in 1996, to evaluate the relationship between risk management practices and the financial performance of 24 Ethiopian MFIs from 2013 to 2023.

Compared to the existing literature, the authors offer a comprehensive view of the risks affecting MFIs’ management practices, over a relatively extended period, and integrating the regulatory context in the risk analysis.

I found that there are some notable contributions and potential new aspects of this research:

1. Comprehensive Risk Management Analysis. The study examines a broad range of risks affecting MFIs’ management practices. Prior research often focused predominantly on credit risk. Hence, including a wider spectrum of (strategic, credit, liquidity, interest rates, and operational) risks provides a more holistic view of the factors influencing MFI performance.
2. Regulatory Context: The study considers the impact of Ethiopia's microfinance legislation introduced in 1996 and subsequent regulatory developments. Understanding how regulatory changes affect MFIs' risk management and performance is a relatively novel aspect, as most studies do not integrate the regulatory dimension deeply.
3. Methodological Approach. By challenging the mixed and inconsistent findings of previous research on the relationship between risk management and MFIs’ performance, the methodology and the specific analytical techniques used in this study could offer new perspectives or validate findings with stronger empirical evidence.

***

MAIN CONCERNS

Below are some potential areas that might be missing or could benefit from further exploration:

1. Comparative Analysis with Other Regions:

While the focus on Ethiopian MFIs provides important insights, a comparative analysis with MFIs in other countries or regions could offer a broader perspective and help identify unique or common challenges and best practices.

2. Client Impact Assessment:

The study focuses on the financial performance of MFIs, but it might not thoroughly assess the impact of MFIs on their clients, especially the socio-economic outcomes for low-income households and women. Evaluating (or at least discussing) the direct benefits to clients could provide a more comprehensive view of MFI effectiveness.

4. Technology and Innovation in MFIs:

The role of technological advancements and financial innovations (e.g., mobile banking, fintech solutions) in improving risk management and financial performance is not taken into consideration. Exploring/discussing how these technologies are integrated into Ethiopian MFIs could be valuable.

5. Detailed Examination of External Factors:

The study should benefit from fully accounting for external factors such as economic conditions, political stability, and social dynamics that can influence MFIs’ performance and risk management practices. A more detailed analysis and inclusion of these external influences could enhance the study's findings. Furthermore, assessing environmental, social (e.g., ESG), and governance factors could provide a more comprehensive view of MFI performance. Finally, the influence of local cultural and behavioral factors on risk management practices and MFI performance should be thoroughly examined.

***

OTHER CONCERNS

- Data Period and Sample Size. The paper uses data collected from 24 MFIs over a decade (2013 to 23). This extended period might offer robust and reliable insights in the medium run, compared to other studies focusing on shorter time frames. However, the sample size is very small (24 MFIs); I suggest adding power tests.
- Practical Implications for Policy and Practice. The study’s findings could have significant implications for policymakers, MFI managers, and stakeholders, although confined to Ethiopia. I suggest discussing more in-depth the external validity of the paper’s findings, clearly indicating what can enhance the financial performance and sustainability of MFIs in other contexts.
