# Supplementary material for: Association of risk management practices and financial performance of microfinance institutions in Ethiopia: A two-step system generalized method of moments approach
Source: PLoS One. 2025 Jul 10;20(7):e0321415. doi: 10.1371/journal.pone.0321415 (PMC12244555; doi:10.1371/journal.pone.0321415)
Supplement: Attachment — Submitted filename: Response to Reviewers.docx [file pone.0321415.s007.docx]

**A rebuttal letter that responds to each point raised by the academic editor and reviewer(s)**
Name of the authors: Megbaru Tesfaw Molla and Ratinder Kaur
Department: School of Management Studies, Punjabi University, Patiala, India
Title: ASSOCIATION OF RISK MANAGEMENT PRACTICES AND FINANCIAL PERFORMANCE OF MICROFINANCE INSTITUTIONS IN ETHIOPIA
Manuscript ID: PONE-D-24-16639

Date: 17/12/2024

Dear Patrick Goymer,

Staff Editor PLOS ONE

Thank you for the opportunity to revise our manuscript titled “**ASSOCIATION OF RISK MANAGEMENT PRACTICES AND FINANCIAL PERFORMANCE OF MICROFINANCE INSTITUTIONS IN ETHIOPIA”** and for the thoughtful comments and suggestions provided by the reviewers. We greatly appreciate your time and also, we thank the reviewers for their time and effort, as the feedbacks have helped to improve the clarity, depth, and overall quality of the paper. Below, we provide a detailed point-by-point response to the comments raised. For clarity, comments are presented in **bold**, followed by our responses in plain text. All changes made in the manuscript are highlighted in **red color.** We trust that the revisions and clarifications made in response to the comments have strengthened the manuscript. If you have any further questions or require additional modifications, we would be happy to address them promptly.

Thank you once again for your constructive feedback and consideration of our work.

Sincerely,

Megbaru Tesfaw Molla
email: [tmegbaru@gmail.com](mailto:tmegbaru@gmail.com)

Cell phone: +251969013317

**Reviewer 1 Comments**

1. **Comment 1:** Comparative Analysis with Other Regions: While the focus on Ethiopian MFIs provides important insights, a comparative analysis with MFIs in other countries or regions could offer a broader perspective and help identify unique or common challenges and best practices.

**Response:** Thank you for this insightful comment. We agree that a comparative analysis with microfinance institutions (MFIs) in other countries or regions would enhance the broader applicability and contextual understanding of our findings. But, in this time, we can’t address this comparison since it needs additional data and more time to collect and analyze data.

2. **Comment 2:** Client Impact Assessment: The study focuses on the financial performance of MFIs, but it might not thoroughly assess the impact of MFIs on their clients, especially the socio-economic outcomes for low-income households and women. Evaluating (or at least discussing) the direct benefits to clients could provide a more comprehensive view of MFI effectiveness.

**Response:** Thank you for your thoughtful comment regarding the importance of assessing the socio-economic outcomes of microfinance institutions (MFIs) on clients. While this study primarily focuses on the financial performance of MFIs, we appreciate your perspective on exploring client-level impacts as an additional dimension. But, the scope of this study limits us to see only the association of risk management practices on financial performance of MFIs in Ethiopia.

3. **Comment 4:** Technology and Innovation in MFIs: The role of technological advancements and financial innovations (e.g., mobile banking, fintech solutions) in improving risk management and financial performance is not taken into consideration. Exploring/discussing how these technologies are integrated into Ethiopian MFIs could be valuable.
   **Response:** Thank you for your insightful comment regarding the role of technology and innovation in microfinance institutions (MFIs). While the primary focus of this study is on the financial performance of Ethiopian MFIs, we agree that technological advancements like mobile banking and fintech solutions play an increasingly significant role in shaping the sector. Therefore, we regret to expand the scope of this study to technological and innovation in MFIs.
4. **Comment 5:** Detailed Examination of External Factors: The study should benefit from fully accounting for external factors such as economic conditions, political stability, and social dynamics that can influence MFIs’ performance and risk management practices. A more detailed analysis and inclusion of these external influences could enhance the study's findings. Furthermore, assessing environmental, social (e.g., ESG), and governance factors could provide a more comprehensive view of MFI performance. Finally, the influence of local cultural and behavioral factors on risk management practices and MFI performance should be thoroughly examined.

**Response:** Thank you for your thoughtful and detailed comment about the role of external factors in influencing the performance and risk management practices of MFIs. While this study primarily focuses on the association of different risk with financial performance within the context of internal operations, we appreciate your suggestion to consider broader economic, political, and social dynamics, as well as ESG factors. These elements indeed provide valuable perspectives for understanding the multifaceted nature of MFI performance.

5. **OTHER CONCERNS**
   • Data Period and Sample Size. The paper uses data collected from 24 MFIs over a decade (2013 to 23). This extended period might offer robust and reliable insights in the medium run, compared to other studies focusing on shorter time frames. However, the sample size is very small (24 MFIs); I suggest adding power tests.

**Response:** We recognize the significance of expanding the sample size to enhance the robustness of the study. However, in the context of Ethiopian Microfinance Institutions (MFIs), this is constrained by the available data. Out of the 34 MFIs operating in Ethiopia, 10 have commenced operations only recently and therefore lack the requisite 10 years of financial data (2013–2023). Consequently, we are limited to considering only those MFIs that have been operational since 2013. Based on this criterion, 24 MFIs qualify to be included in the sample, ensuring consistency and reliability in the data used for analysis.

• Practical Implications for Policy and Practice. The study’s findings could have significant implications for policymakers, MFI managers, and stakeholders, although confined to Ethiopia. I suggest discussing more in-depth the external validity of the paper’s findings, clearly indicating what can enhance the financial performance and sustainability of MFIs in other contexts.

**Response:** We acknowledge the importance of this point and have taken the following steps to address it: We have pointed out the practical implications for policy and Practices. The revised text and practical implications for policy and Practices page found 23.

**Reviewer 2 Comments**

1. **Comment 1:** Title and theme of the paper is justified. But title needs revision. Make a scientific title following the call for paper of this journal.

**Response:** Thank you for your positive feedback on the title and theme of the paper. While the current title aligns with the core focus of the study, we appreciate your suggestion to ensure it aligns closely with the journal's call for papers.

2. **Comment 2:** Overall structure of thematic content is OK. In my opinion, authors have taken a limited approach to measure and study cleaner environment. Authors must take a multidimensional approach to study natural resources and green economic recovery that need to be improved.

**Response:** Thank you for your constructive feedback on the structure of the thematic content. We are pleased to know that you find the overall structure satisfactory. While this study takes a focused approach to examining the association of risk management practices and financial performance of MFIs, your insights highlight valuable directions for expanding the scope of future research to capture a more holistic understanding of these interconnected themes.

3. **Comment 3:** The abstract of the manuscript is defined and precise but still there is a room to improve it. However, it should discuss the finding of the study along with policy implications in a way that reader generates interest to read the whole paper. If possible, please add 1-2 lines about policy implications in the abstract.

**Response:** Thank you for your thoughtful feedback on the abstract. We appreciate your suggestion to enhance it by including the study's findings and policy implications to make it more engaging for readers. We have carefully considered your input and have revised the abstract to include 1-2 lines about the policy implications, aiming to highlight the study's practical relevance and encourage readers to explore the full paper as it is highlighted in red color in page 1.

4. **Comment 4:** Introduction section requires to explain the novelty of the paper including study motivation, contribution and research problem. Please try to revise the contribution and study motivation paragraph with brief and comprehensive detail.
   **Response:** We acknowledge the importance of this point and have taken the following steps to address it: We have tried to show the nobility of the study, motivations and its contributions well. The revised text can be found in page 2 and 3 highlighted with red color.
5. **Comment 5:** The contribution seems dim in this manuscript. I would suggest the author to enhance your theoretical discussion and arrives your debate or argument to show satisfactory contribution. Refine research contribution and objectives. Clearly state both theoretical and practical contributions.

**Response:** We have accepted this comment and we have pointed out the practical implications for policy and Practices and make the conclusion specific to each responsible body of MFIs. The revised text and practical implications for policy and Practices page found 23.

6. **Comment 6:** The authors have failed to explain the gap for the current study, although on page 6 they have mentioned a brief summary, but just naming some techniques is not sufficient to highlight the importance of current study. Please revise

**Response:** Thank you for your insightful observation. You bring up a valid point regarding the explanation of the gap for the current study. We appreciate your constructive feedback, and we agree that a revision in this section could greatly improve the paper and we included a section 2. Research Gap on page 6 of the revised manuscript.

7. **Comment 7:** Literature review section is finely attempted but this section still need improvement. I think the authors must revisit literature again and re-write in fine academic way. Because as in its present state it is not sufficient.

**Response:** We acknowledge the importance of this point and have taken the following steps to address it: We revisited the literature review part and tried to add some literature. The revised texts are highlighted on red color and found on page 5 and 6 of the revised manuscript.

8. **Comment 8:** Overall, the methodology of study looks fine, Rectify syntax errors and grammatical issues in it. Particularly, you can improve the advantages of this and its application with broader context.

**Response:** We acknowledge the importance of this point and we have revisited to correct grammatical errors and tried to refine the methodology. The revised sections are highlighted in red color.

9. **Comment 10:** Overall, results section is also fine. Add rigor in this section and present some graphs in this section. Add some more options to develop rigor in results and discussion section. In results sections, link your study results with the previous studies. Give a comparison of findings in your results study. Make sequential orders of all results sections also cite table numbers in it. Add some beautiful graphs too.

**Response:** We acknowledge the importance of this point and we have revised the result section and supported the results with findings of previous studies and results are compared accordingly. The chanced made are highlighted in red color.

10. **Comment 11:** Suitable captions and heading title of the figures and tables are needed before acceptance of the paper.

**Response:** We acknowledge this comments and we tried to clear the titles and figures, which are essential for understanding their content and purpose. The revised title and headings are highlighted in red color.

11. **Comment 13:** The policy response of different countries should be explained in detail along with the policy recommendations.

**Response:** We acknowledge the importance of this point and we have considered the policy implication of Ethiopia based on the result of the study and tried to compare with other countries policy.

**Reviewer 3 Comments**

1. **Comment 1:** Research Relevance and Contribution: While the topic of risk management practices and financial performance of microfinance institutions (MFIs) is of importance, the article does not sufficiently clarify its contribution to the existing literature. It lacks a thorough review of previous studies to establish the novelty of the research and its implications for policymakers and practitioners.

**Response:** Thank you for pointing this out. In response, we included a review of recent published papers at the end of the literature review on page 5 and 6 of the revised manuscript. We have also showed its contributions to the existing literature and its implications for policymakers and practitioners, the revised point is found at the end of the introduction on page 3 and at the recommendation part on page 23.

2. **Comment 2:** Methodology Issues: The study employs a mixed research approach; however, further details are necessary to understand how qualitative data were integrated with quantitative findings. The methodological rigor appears to be inadequate due to the absence of clear justification for the selection of the GMM estimator and the sample size. The decision-making process regarding the selection of the 24 MFIs should be better explained to enhance reproducibility.

**Response:** Regarding the mixed approach, we have wrongly stated in the abstract part, but in the methodology part it is correctly stated as the study employed explanatory research design and only quantitative data has been used in this study. Regarding the justification of GMM estimator, we have accepted the comment and justified it as it is highlighted in red color on page 9 of the revised manuscript.. We recognize the significance of expanding the sample better explained to enhance reproducibility. However, in the context of Ethiopian Microfinance Institutions (MFIs), it is constrained by the available data. Out of the 34 MFIs operating in Ethiopia, 10 have commenced operations only recently and therefore lack the necessary 10 years of financial data (2013–2023). Consequently, we are limited to considering only those MFIs that have been operational since 2013. Based on this criterion, 24 MFIs qualify to be included in the sample, ensuring consistency and reliability in the data used for analysis.

3. **Comment 3:** Data Collection and Analysis: The article mentions secondary data collection from financial statements, but it does not discuss the methods used to ensure the reliability and validity of this data. Additionally, while the statistical analysis with STATA is noted, there is insufficient detail on how the analysis controlled for potential confounding variables and biases.

**Response:** We acknowledge the importance of this point and we have mentioned the methods we have used to ensure the reliability and validity of secondary data used. In addition, we have justified the reasons of using STATA statistical analysis. The changes made are highlighted with red color and found on page 8 of the revised documents.

4. **Comment 4:** Findings and Interpretations: The findings highlight important relationships, but there is a lack of depth in the interpretation of these results. For instance, the report of negative relationships should explore potential reasons behind these phenomena. Also, the implications of non-significant relationships, particularly concerning nonperforming loans and performance indicators, warrant further exploration.

**Response:** We acknowledge the importance of this point and we have tried to justify interpretation of these results among the variables as it is highlighted in red color.

5. **Comment 5:** Presentation and Clarity: The writing lacks clarity in some sections, making it challenging to follow the argumentation. More precise language and structure are needed to improve comprehension. Additionally, several technical terms are used without adequate explanation, which could alienate readers unfamiliar with the subject matter.
   **Response:** We acknowledge the importance of this we have revised the article and go through the language correction. Regarding technical terms, we have included a section 2.4. variable definition and measurement which is highlighted in red color and found on page 10 up to page 12 of the revised manuscript.
6. **Comment 6:** Conclusion and Recommendations: The conclusion seems to overgeneralize the findings without providing actionable recommendations. It would enhance the article to include suggestions for practitioners in the microfinance sector based on the study's result

**Response:** We have accepted this comment and we have pointed out the practical implications for policy and Practices and make the conclusion specific to each responsible body of MFIs. The revised text and practical implications for policy and Practices page found 23.

7. **Comment 7** References and Citations: The references cited in the article are not extensive and seem outdated in some areas. An updated literature review would bolster the research's credibility and context.

**Response:** We acknowledge the importance of this point and we have reviewed and included recently published articles to bolster the research's credibility and context. The revised articles found on page 5 and 6 of the revised manuscript.
