# Supplementary material for: Association of risk management practices and financial performance of microfinance institutions in Ethiopia: A two-step system generalized method of moments approach
Source: PLoS One. 2025 Jul 10;20(7):e0321415. doi: 10.1371/journal.pone.0321415 (PMC12244555; doi:10.1371/journal.pone.0321415)
Supplement: Attachment — Submitted filename: PONE-D-24-16639_Editor_R1.docx [file pone.0321415.s005.docx]

**Referee report for**

PONE-D-24-16639_R1
TITLE: ASSOCIATION OF RISK MANAGEMENT PRACTICES AND FINANCIAL PERFORMANCE OF MICROFINANCE INSTITUTIONS IN ETHIOPIA

**To the Editor**

Dear Editor,

Thanks for allowing me to further review this article.

In my previous report I stated that the paper could provide some notable contributions to the literature on microfinance in developing contexts. However, few or none of the issues I raised in my report were taken into consideration by the authors. Consider that some did not even require a particular effort in terms of information and data collection. Therefore, I do not understand the preponderance of rebuttals on the issues I raised.

As highlighted in my first report, potential new aspects of this research could make the paper interesting for a wider audience and not confined to the specific issues of Ethiopia. However, I find that in the new version of the paper the Authors are even more focused on the specific Ethiopian context, especially in the Introduction.

Furthermore, with reference to methodological issues, I suggested conducting robustness tests to support the use of a very limited number of MFIs (24). I do not find it justifiable that the authors avoided conducting trivial power tests.

Consequently, I do not believe the paper is publishable in its current state.

Best regards,

Lucia Dalla Pellegrina
