# Supplementary material for: Association of risk management practices and financial performance of microfinance institutions in Ethiopia: A two-step system generalized method of moments approach
Source: PLoS One. 2025 Jul 10;20(7):e0321415. doi: 10.1371/journal.pone.0321415 (PMC12244555; doi:10.1371/journal.pone.0321415)
Supplement: Attachment — Submitted filename: PONE-D-24-16639_Authors_R1.docx [file pone.0321415.s006.docx]

**Referee report for**

PONE-D-24-16639_R1
TITLE: ASSOCIATION OF RISK MANAGEMENT PRACTICES AND FINANCIAL PERFORMANCE OF MICROFINANCE INSTITUTIONS IN ETHIOPIA

**To the Authors**

Unfortunately, few or none of the issues I raised in my previous report were taken into consideration, some did not even require a particular effort in terms of information and data collection. Therefore, I do not understand the preponderance of rebuttals on the issues I raised.

As highlighted in my first report there are some notable contributions and potential new aspects of this research that could make the paper more interesting for a wider audience and not confined to the specific issues of Ethiopia. This is a fundamental aspect that I believe cannot be bypassed.

In particular:

“*Comparative analysis with MFIs in other countries or regions could offer a broader perspective and help identify unique or common challenges and best practices”.* The authors provide a rebuttal that does not make much sense. Based on their experience in microfinance, and their knowledge of the specific context of Ethiopia, they could provide useful indications in terms of exportability of the results obtained in other developing countries that have similar institutional conditions to Ethiopia. This can be commented on without necessarily resorting to further data collection. However, if this were necessary, one could rely on synthetic and aggregate indicators developed by platforms such as MIX Market, with minimal effort in terms of processing and comparison.

“*The role of technological advancements and financial innovations (e.g., mobile banking, fintech solutions) in improving risk management and financial performance is not taken into consideration. Exploring/discussing how these technologies are integrated into Ethiopian MFIs could be valuable*.” The authors argue that they regret to expand the scope of this study to technological and innovation in MFIs without even providing an argument in this regard. I ask them not to underestimate this aspect, at least try to provide some reflections in the conclusions.

“*Data Period and Sample Size. The paper uses data collected from 24 MFIs over a decade (2013 to 23). This extended period might offer robust and reliable insights in the medium run, compared to other studies focusing on shorter time frames. However, the sample size is very small (24 MFIs); I suggest adding power tests.*” I understand that the authors are constrained by the availability of data on 24 MFIs only. But avoiding providing power tests is not justifiable.
