# Supplementary material for: Association of risk management practices and financial performance of microfinance institutions in Ethiopia: A two-step system generalized method of moments approach
Source: PLoS One. 2025 Jul 10;20(7):e0321415. doi: 10.1371/journal.pone.0321415 (PMC12244555; doi:10.1371/journal.pone.0321415)
Supplement: Attachment — Submitted filename: Response_to_Reviewers_auresp_2.docx [file pone.0321415.s009.docx]

**A rebuttal letter that responds to each point raised by the academic editor and reviewer(s)**
Name of the authors: Megbaru Tesfaw Molla and Ratinder Kaur
Department: School of Management Studies, Punjabi University, Patiala, India
Title: ASSOCIATION OF RISK MANAGEMENT PRACTICES AND FINANCIAL PERFORMANCE OF MICROFINANCE INSTITUTIONS IN ETHIOPIA
Manuscript ID: PONE-D-24-16639

Date: 12/05/2025

Dear Kennedy Munyua Waweru, PhD

Academic Editor PLOS ONE

Thank you for the opportunity to revise our manuscript titled “**ASSOCIATION OF RISK MANAGEMENT PRACTICES AND FINANCIAL PERFORMANCE OF MICROFINANCE INSTITUTIONS IN ETHIOPIA”** and for the thoughtful comments and suggestions provided by the reviewers. We greatly appreciate your time and also, we thank the reviewers for their time and effort, as the feedbacks have helped to improve the clarity, depth, and overall quality of the paper. Below, we provide a detailed point-by-point response to the comments raised. For clarity, comments are presented in **bold**, followed by our responses in plain text. All changes made in the manuscript are highlighted in **red color.** We trust that the revisions and clarifications made in response to the comments have strengthened the manuscript. If you have any further questions or require additional modifications, we would be happy to address them promptly.

Thank you once again for your constructive feedback and consideration of our work.

Sincerely,

Megbaru Tesfaw Molla
email: [tmegbaru@gmail.com](mailto:tmegbaru@gmail.com)

Cell phone: +251912089760

**Comments and responses**

1. **Comment 1:** “*Comparative analysis with MFIs in other countries or regions could offer a broader perspective and help identify unique or common challenges and best practices”.* The authors provide a rebuttal that does not make much sense. Based on their experience in microfinance, and their knowledge of the specific context of Ethiopia, they could provide useful indications in terms of exportability of the results obtained in other developing countries that have similar institutional conditions to Ethiopia. This can be commented on without necessarily resorting to further data collection. However, if this were necessary, one could rely on synthetic and aggregate indicators developed by platforms such as MIX Market, with minimal effort in terms of processing and comparison.

**Response:** Thank you for this insightful comment. We appreciate your emphasis on enhancing the broader relevance of our study. While our original focus was on Ethiopian MFIs, we now recognize the value of discussing the *exportability* of our findings. Accordingly, we have added a comparative reflection section in the discussion and conclusion parts of the paper. This includes brief comparative insights using publicly available aggregate indicators from MIX Market Database. We also highlight shared challenges such as interest rate risk and loan default management that are common across MFIs in sub-Saharan Africa, South Asia, and other developing economies with similar institutional frameworks. This has been done without expanding the scope of primary data collection, as per your guidance.

2. **Comment 2:** “*The role of technological advancements and financial innovations (e.g., mobile banking, fintech solutions) in improving risk management and financial performance is not taken into consideration. Exploring/discussing how these technologies are integrated into Ethiopian MFIs could be valuable*.” The authors argue that they regret to expand the scope of this study to technological and innovation in MFIs without even providing an argument in this regard. I ask them not to underestimate this aspect, at least try to provide some reflections in the conclusions.

**Response:** Thank you for pointing out this important gap. While this study does not directly analyze technology-related variables due to data limitations, we agree that fintech and digital tools play an increasingly central role in microfinance. We appreciate your suggestion and have included reflections on the potential influence of such technologies in the revised conclusion section. Future research is indeed encouraged to investigate how Ethiopian MFIs are integrating these innovations and the resulting implications for sustainability and financial resilience. This will allow for a more holistic understanding of performance dynamics in the sector.

3. **Comment 3:** “Data Period and Sample Size. The paper uses data collected from 24 MFIs over a decade (2013 to 23). This extended period might offer robust and reliable insights in the medium run, compared to other studies focusing on shorter time frames. However, the sample size is very small (24 MFIs); I suggest adding power tests.” I understand that the authors are constrained by the availability of data on 24 MFIs only. But avoiding providing power tests is not justifiable.

**Response:** We fully acknowledge the concern regarding the limited sample size of 24 MFIs. As you correctly noted, the ten-year data span (2013–2023) was intended to enhance the robustness and reliability of the analysis by leveraging the dynamic nature of the panel dataset. In response to your suggestion, we have conducted and included a power test for the cross-sectional component of the analysis. The test, based on a sample size of 24 MFIs, 10 predictors, a medium effect size (f² = 0.15), and a significance level of α = 0.05, indicated a power of approximately 5.37%, which is well below the conventional threshold of 80%. This confirms the study’s limited ability to detect moderate effects in the cross-sectional analysis. To address this limitation, we employed a dynamic panel data model (GMM), which utilizes within-entity variation over time and enhances estimation efficiency. Nonetheless, we have explicitly acknowledged the power limitation and cautioned readers regarding the interpretation of cross-sectional results. We have also emphasized the need for future research using larger and more diverse samples to strengthen generalizability.

4. **Comment 4:**  the author(s) need to explain more on the implication of this study on how the results benefiting by the related parties.

**Response:** Thank you for the constructive feedback. In response to the comment regarding the need to explain more on the implications of the study, we have revised the manuscript accordingly. We have added a detailed explanation in the implications to highlight how the findings can benefit the relevant stakeholders and related parties.

5. **Comment 4:**  Please review your reference list to ensure that it is complete and correct. If you have cited papers that have been retracted, please include the rationale for doing so in the manuscript text, or remove these references and replace them with relevant current references. Any changes to the reference list should be mentioned in the rebuttal letter that accompanies your revised manuscript. If you need to cite a retracted article, indicate the article’s retracted status in the References list and also include a citation and full reference for the retraction notice.

**Response:** Thank you for your comment on the reference list. We have reviewed and corrected some the misused references. All changes have been incorporated into the revised manuscript.

We hope these changes address your concerns fully and enhance the scholarly contribution and international relevance of the paper.

With sincere appreciation,

Megbaru Tesfaw Molla
