# Supplementary material for: Association of risk management practices and financial performance of microfinance institutions in Ethiopia: A two-step system generalized method of moments approach
Source: PLoS One. 2025 Jul 10;20(7):e0321415. doi: 10.1371/journal.pone.0321415 (PMC12244555; doi:10.1371/journal.pone.0321415)
Supplement: Attachment — Submitted filename: PONE-D-24-16639_Editor_R2.docx [file pone.0321415.s008.docx]

**Referee report for**

**PONE-D-24-16639_R2
TITLE: ASSOCIATION OF RISK MANAGEMENT PRACTICES AND FINANCIAL PERFORMANCE OF MICROFINANCE INSTITUTIONS IN ETHIOPIA**

**To the Editor**

Dear Editor,

The authors have addressed all the comments raised in the previous round of review in a clear and satisfactory manner. The revised manuscript now presents a well-articulated research question, a more robust theoretical framework, and a clearly described methodology. The rebuttal letter responds to each point systematically, and the language and presentation have been significantly improved. I recommend the manuscript for publication in its current form or with only minor editorial adjustments, if needed.

Sincerely,

Lucia dalla Pellegrina
